# Evolutionary potentials: structure specific knowledge-based potentials exploiting the evolutionary record of sequence homologs

**DOI:** 10.1186/gb-2008-9-4-r68

**Published:** 2008-04-08

**Authors:** Alejandro Panjkovich, Francisco Melo, Marc A Marti-Renom

**Affiliations:** 1 Departamento de Genética Molecular y Microbiología, Facultad de Ciencias Biológicas, Pontificia Universidad Católica de Chile, Alameda 340, Santiago, Chile; 2 Structural Genomics Unit, Bioinformatics Department, Centro de Investigación Príncipe Felipe (CIPF), Av. Autopista del Saler, 16, 46013 Valencia, Spain; 3 Current address: Institute for Research in Biomedicine (IRB) and Barcelona Supercomputing Center (BSC), c/Josep Samitier 1-5, 08028 Barcelona, Spain

## Abstract

We introduce a new type of knowledge-based potentials for protein structure prediction, called 'evolutionary potentials', which are derived using a single experimental protein structure and all three-dimensional models of its homologous sequences. The new potentials have been benchmarked against other knowledge-based potentials, resulting in a significant increase in accuracy for model assessment. In contrast to standard knowledge-based potentials, we propose that evolutionary potentials capture key determinants of thermodynamic stability and specific sequence constraints required for fast folding.

## Abstract 2

So-called ‘Evolutionary potentials’ for protein structure prediction are derived using a single experimental protein structure and all three-dimensional models of its homologous sequences.

## Background

Comparative protein structure prediction is typically implemented in four main steps: fold assignment, target-template alignment, model building, and model assessment [1]. Thus, the starting point in comparative modeling identifies protein structures related to the target sequence. This initial step is normally performed using profile-based search methods such as PSI-BLAST, hidden Markov models or profile-profile methods [2]. Once a fold has been assigned, a specialized alignment method is used to optimally align the target sequence with the template structure. Then, the target-template alignment is used to build a structure model of the target sequence. Finally, the model assessment step predicts whether or not the correct template was assigned and at least an approximately correct alignment was produced. Thus, estimating the accuracy of a protein structure model is essential for determining the information that can be extracted from it. In this work we describe the development and implementation of a new method for model assessment.

Physics and knowledge-based scoring functions, which comprise an essential tool for computationally predicting the three-dimensional structure of a polypeptide, are now routinely applied in model assessment. Two fundamentally different approaches have been developed for deriving such scoring functions. The first approach, which is of inductive nature, uses simplified mathematical models for describing the system without previous knowledge of its properties [3]. The second approach, which is of deductive nature, uses geometrical descriptors derived from known protein structures to score the interaction between two or more particles [4]. These types of scoring functions, which are often referred to as statistical potentials, knowledge-based potentials or potentials of mean force, have previously been applied in many different assessment problems, including: determination of whether or not a model has the correct fold [5-8]; detection of localized errors in protein structures [9]; assessment of the stability of mutant proteins [10]; discrimination between native and near-native states [11-13]; and selection of the most near-native model in a set of decoys that does not contain the native structure [13-15]. In general, different types of assessment problems can benefit from specialized scoring functions or classifiers [16]. In this work, we focus on the model assessment problem that assesses whether or not a given model has the correct fold and was built using an approximately correct alignment [17,18].

Standard knowledge-based potentials are derived by applying the inverse of the Boltzmann's equation on distributions of geometrical features calculated from a non-redundant set of known protein structures [4]. Therefore, such potentials effectively capture the general trends of atomic interactions for average globular proteins. However, each protein structure may have specific features that are critical for its folding and stability, which are either not captured or poorly represented by current knowledge-based potentials. In this work, we describe a methodology to derive a new type of knowledge-based potentials, here termed evolutionary potentials (EvPs), which is designed to overcome such a limitation. EvPs are specifically derived for a restricted structural space based on a single experimental structure and all its detectable protein sequence homologues. We propose that the incorporation of a large amount of sequence information mapped onto a restricted three-dimensional space allows EvPs to capture and balance the specific key interactions occurring within a given protein fold.

We begin this article by assessing the accuracy of the EvPs in model assessment, comparing it to a representative potential of identical parameterization as well as to other commonly used knowledge-based potentials (Results). Next, we discuss the key determinants in the implementation of EvPs as well as their likely impact on protein structure prediction of genes and genomes. Finally, we end by describing the proposed methodology for the derivation of EvPs (Materials and methods).

## Results

EvPs are calculated using a single experimental structure and the threaded models of its detectable homologous sequences, which represent the space of protein sequences that may adopt its fold. Thus, two major variables impact the derivation of EvPs: the stringency of the clustering process (that is, the structural clustering cut-offs) and the deepness of the multiple sequence alignments (MSAs) (that is, the sequence identity cut-off for selecting homologous sequences). First, we assess the impact of these two variables on the accuracy of the EvPs for model assessment. Second, we assess the impact of EvP selection in the same benchmark. Finally, we compare the accuracy of EvPs to that obtained with a representative distance-dependence potential derived using the same parameters, and also with other potentials such as DFIRE [19] and Prosa II [4,20,21].

### Strict structural clustering results in more accurate EvPs

**Figure 1:**
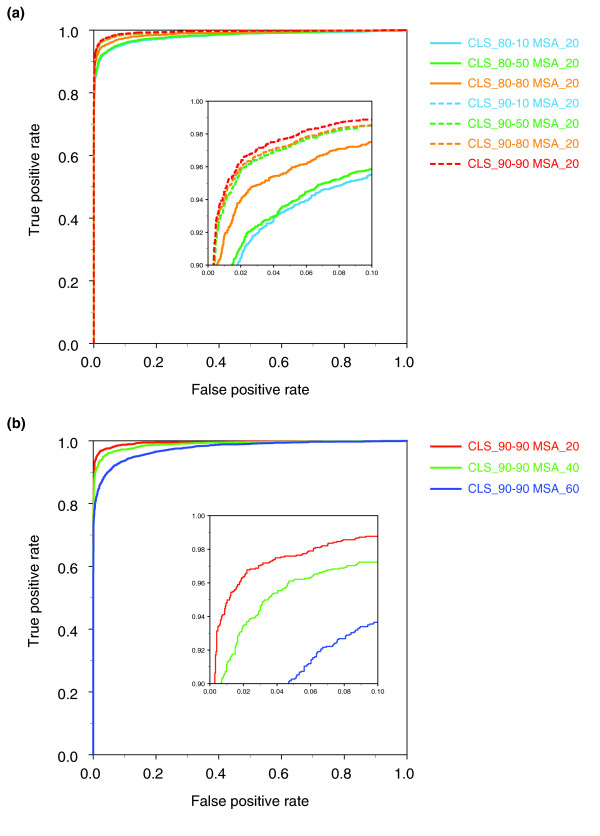
EvP accuracy at different structure and sequence clustering cut-offs. **(a) **ROC curves for the different EvP sets depending on the structural clustering of the PDB space. The inner panel zooms into the upper-left corner of the ROC curve to better show the differences between the curves. **(b) **ROC curves for the different EvP sets derived using different cut-offs of sequence identity in the MSA. The inner panel zooms into the upper-left corner of the ROC curve to better show the differences between the curves.

The clustering of the structural space affects the selection and specificity of EvPs for model assessment. Various combinations of thresholds for structure similarity (that is, 80% and 90% of Cα atoms within 4 Å) and sequence identity (that is, 90%, 80%, 50%, 20%, and 10%) were applied to obtain 10 different sets of representative chains. EvPs calculated from the strictest clustering corresponding to 90% sequence and structural identity resulted in the most accurate assessment of the model accuracy as measured by their maximal accuracy (ACC), the area under the curve (AUC), false positive rate (FPR), and true positive rate (TPR) (that is, 99.5% AUC, 97.4% ACC, 2.3% FPR, and 97.0% TPR; Table 1). Variation of sequence identity for clustering had a marginal impact on the accuracy of the EvPs. However, a decrease of only 10% in the cut-off for the structural similarity had a larger impact, reducing the ACC and the TPR of the EvPs up to approximately 2% and 4%, respectively. Therefore, the accuracy of the EvPs for model assessment decreases as more structural variability is allowed within the clusters that represent the structure space (Table 1; Figure 1a; Table S1 in Additional data file 1).

**Table 1:** EvP model assessment accuracy for different clustering parameters

EvP	AUC (%)	ACC (%)	OT	FPR (%)	TPR (%)
CLS_90-90 MSA_20	99.5	97.4	-4.18	2.3	97.0
CLS_90-80 MSA_20	99.3	97.2	-4.18	2.1	96.2
CLS_90-50 MSA_20	99.0	97.0	-4.18	2.2	95.8
CLS_90-10 MSA_20	99.0	97.0	-4.18	2.1	95.8
CLS_80-80 MSA_20	98.8	96.5	-4.18	1.8	94.2
CLS_80-50 MSA_20	97.7	94.7	-3.99	2.2	90.4
CLS_80-10 MSA_20	97.6	94.4	-3.92	2.5	90.2

The sets of EvPs are named according to the clustering parameters (CLS_XX-YY) and the multiple sequence alignment filters (MSA_ZZ) used to derive them (Materials and methods). XX is the minimal structural similarity and YY is the minimal sequence identity used for structural clustering. ZZ is the minimal sequence identity shared between a sequence in the multiple sequence alignment and the sequence of the representative structure of the cluster. AUC is the area under the ROC curve, ACC is the maximal accuracy, OT is the optimal classification threshold, FPR is the false positive rate, and TPR is the true positive rate.

### Incorporation of more distantly related sequences results in more accurate EvPs

The selected sequence space (that is, sequences in the MSA) used for the derivation of EvPs affects their accuracy for model assessment. EvPs derived using 20%, 40%, and 60% cut-offs for homology detection were calculated for 88.2%, 59.5%, and 31.2% of all non-redundant chains, respectively (Table S2 in Additional data file 1). The remaining EvPs were not calculated because the input MSA at the respective sequence identity cut-off contained less than 50 sequences. In those cases, the number of sequences to thread is too small to reliably derive a potential [18].

The accuracy of the EvPs upon the selected sequence space shows an opposite trend to that observed for the structural clustering (Table 2; Figure 1b; Table S3 in Additional data file 1). The filtering cut-off of 20% sequence identity results in 97.4% ACC and 99.5% AUC. Increasing the sequence identity cut-off results in an accuracy decrease of the EvPs. Thus, the amount and similarity of the input sequences to derive the EvPs have an impact on their accuracy. The average sequence identity between the template and its homologous sequences in the MSAs used for deriving EvPs was approximately 30% (Table S2 in Additional data file 1), which indicates that EvPs are able to capture relevant information even from distantly related sequences. The difference in the resulting accuracy decreases as we approach the limits of homology detection (Figure 1b). Thus, the increase in accuracy by lowering the sequence identity cut-off from 60% to 40% is larger than that observed when lowering it from 40% to 20%.

**Table 2:** EvP model assessment accuracy for different MSA filtering parameters

EvP	AUC (%)	ACC (%)	OT	FPR (%)	TPR (%)
CLS_90-90 MSA_20	99.5	97.4	-4.18	2.3	97.0
CLS_90-90 MSA_40	99.0	96.1	-4.05	2.0	93.4
CLS_90-90 MSA_60	97.6	93.1	-3.41	4.4	89.7

The sets of EvPs are named according to the clustering parameters (CLS_XX-YY) and the multiple sequence alignment filters (MSA_ZZ) used to derive them (Materials and methods). XX is the minimal structural similarity and YY is the minimal sequence identity used for structural clustering. ZZ is the minimal sequence identity shared between a sequence in the multiple sequence alignment and the sequence of the representative structure of the cluster. AUC is the area under the ROC curve, ACC is the maximal accuracy, OT is the optimal classification threshold, FPR is the false positive rate, and TPR is the true positive rate.

### Strict EvP selection results in more accurate model assessment

The selection of EvPs has an impact on their accuracy for assessing the fold of a structure model. We have used four different strategies for selecting a particular EvP, which consisted of: selection of the EvP that corresponds to the structural representative of the used template for model building; selection of the EvP corresponding to the closest BLAST match using the model sequence against a database of sequences for the calculated EVPs; selection of the EvP corresponding to the closest PSI-BLAST match of the model sequence against a database of sequences for the calculated EVPs; and random selection of an EvP. The first protocol is suitable for a comparative modeling exercise, the second and third protocols are suitable for models built from 'template-free' protein structure prediction methods, and the fourth protocol was used as a negative control for the impact of the EvP selection process.

**Figure 2:**
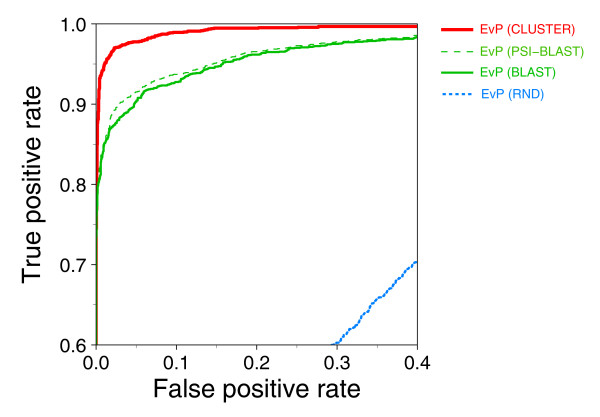
EvP accuracy using different assignment protocols. ROC curves for the three EvP selection protocols: representative EvP of the template used in the model building process (CLUSTER), closest EvP to the target sequence based on a PSI-BLAST search (PSI-BLAST), closest EvP to the target sequence based on a BLAST search (BLAST), and random selection of an EvP (RND). EvP sets in this figure were generated using the CLS_90-90 clustering cut-offs and the MSA_20 MSA filtering cut-off.

The maximal accuracy for model assessment of the EvPs increases with the ability to select the closest EvP to the target sequence (Figure 2; Table S4 in Additional data file 1). Using PSI-BLAST or BLAST for selecting the EvP results in 3.2% and 3.9% lower ACC, respectively, compared to a template-based selection (Table 3). The TPR decreases approximately 10% if the selected EvPs are not structurally close to the real structures of the target proteins. As expected, a random selection of EvPs results in low accuracy (that is, 42.3% TPR and 12.1% FPR), which results in AUC and ACC being 27.6% and 28.8% lower, respectively, than when selecting the EvPs based on the template used for model building (Table 3).

**Table 3:** EvP model assessment accuracy for different selection protocols

Selection protocol	AUC (%)	ACC (%)	OT	FPR (%)	TPR (%)
EvP (CLUSTER)	99.5	97.4	-4.18	2.3	97.0
EvP (PSI-BLAST)	97.7	94.2	-1.86	2.2	89.2
EvP (BLAST)	97.5	93.5	-2.06	1.7	86.9
EvP (RND)	71.9	68.6	-1.82	12.1	42.3

EvPs in this table were generated using the CLS_90-90 clustering cut-offs and the MSA_20 MSA filtering cut-off. The sets of EvPs are named according to the clustering parameters (CLS_XX-YY) and the multiple sequence alignment filters (MSA_ZZ) used to derive them (Materials and methods). XX is the minimal structural similarity and YY is the minimal sequence identity used for structural clustering. ZZ is the minimal sequence identity shared between a sequence in the multiple sequence alignment and the sequence of the representative structure of the cluster. AUC is the area under the ROC curve, ACC is the maximal accuracy, OT is the optimal classification threshold, FPR is the false positive rate, and TPR is the true positive rate.

### EvPs result in the highest accuracy for model assessment

**Figure 3:**
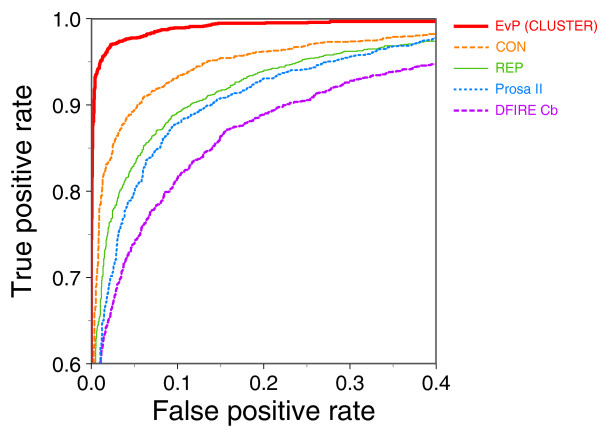
EvP accuracy tested against other knowledge-based potentials. ROC curves for the tested methods: EvP (CLUSTER), consensus EvP potential (CON), representative potential (REP), Prosa potential (Prosa II), and DFIRE potential (DFIRE Cβ). The EvP (CLUSTER) set in this figure was generated using the CLS_90-90 clustering cut-offs and the MSA_20 MSA filtering cut-off.

EvPs outperform the representative potential (REP) with a 7.1% higher ACC, 3.8% lower FPR, and 11.7% higher TPR (Table 4; Figure 3; Table S5 in Additional data file 1). This demonstrates that, even though EvPs are derived for single structures, the incorporation of evolutionary information in the form of MSAs significantly increases the accuracy of distance-dependent potentials for model assessment. Moreover, the use of specific EvPs also outperforms the consensus EvP potential (CON) and other standard knowledge-based potentials such as Prosa II and DFIRE Cβ potentials (Table 4; Figure 3; Table S5 in Additional data file 1). The ACC of EvPs is approximately 7% higher than that of CON, REP, and Prosa II potentials and approximately 11% higher than the DFIRE potential. EvPs also result in a very small FPR (2.3%) and a high TPR (97.3%), being the most sensitive and specific classifier tested in our benchmark for model assessment. The REP and Prosa II potentials, which have been derived using the same principles and differ only in the set of experimental proteins used to derive them, result in very similar accuracies (that is, approximately 95% AUC and approximately 90% ACC). The DFIRE Cβ potential, which not only uses a different training set but is also derived with a different reference state than the REP and Prosa II potentials, results in lower accuracy than any of the other tested methods with a 77.6% TPR and 6.8% FPR.

**Table 4:** EvP model assessment accuracy compared to other potentials

Potential	AUC (%)	ACC (%)	OT	FPR (%)	TPR (%)
EvP (CLUSTER)	99.5	97.4	-4.18	2.3	97.0
CON	97.1	92.8	-2.91	4.1	88.5
REP	95.9	90.3	-2.78	6.1	85.3
Prosa II	95.4	89.4	-2.52	6.3	83.6
DFIRE Cβ	92.7	86.6	-2.76	6.8	77.6

EvPs in this table were generated using the CLS_90-90 clustering cut-offs and the MSA_20 MSA filtering cut-off. The sets of EvPs are named according to the clustering parameters (CLS_XX-YY) and the multiple sequence alignment filters (MSA_ZZ) used to derive them (Materials and methods). XX is the minimal structural similarity and YY is the minimal sequence identity used for structural clustering. ZZ is the minimal sequence identity shared between a sequence in the multiple sequence alignment and the sequence of the representative structure of the cluster. AUC is the area under the ROC curve, ACC is the maximal accuracy, OT is the optimal classification threshold, FPR is the false positive rate, and TPR is the true positive rate. For a detailed description of the CON and REP potentials see Materials and methods.

## Discussion

Generally, the accuracy of a protein structure model (that is, model assessment) can be assessed by physics-based, knowledge-based or a combination of both types of scoring functions. Knowledge-based potentials assess protein structure models by their fitting to the statistical preferences of different residues or atom types to be exposed to the solvent, or to interact with each other in a pairwise or higher order fashion. These statistical preferences are normally extracted from a set of selected structures, which represent the known structural space for native proteins. In this work we have described, implemented and benchmarked a new method for deriving distance-dependent knowledge-based potentials that relies on information from the sequence space of a given structure or fold. The method was validated for model assessment using a large benchmark of 4,444 protein structure models of varying accuracy [7,22]. The results show that the use of homologous sequences for deriving specific knowledge-based potentials increases the specificity and sensitivity for model assessment (Figure 3; supplementary Tables in Additional data file 1). The ACC of the EvPs is 7.1%, 8.0% and 10.8% higher than that of REP, Prosa II, and DFIRE Cβ potentials, respectively. Moreover, EvPs result in very high specificity (that is, a FPR of 2.3%) and sensitivity (that is, a TPR of 97.0%) compared with those for REP, Prosa II, and DFIRE Cβ (that is, FPR > 6%, TPR < 89%). This is particularly relevant for large-scale protein structure prediction, where a small improvement in the TPR and FPR can have a large impact on the total number of models correctly selected and/or wrongly discarded, respectively [17].

The benchmarking of the EvPs against two widely used model assessment methods, Prosa II and DFIRE Cβ, provides a reference line for determining the difficulty of our test set of comparative models. However, it is important to note that neither Prosa II nor DFIRE Cβ were optimized against models generated with the MODELLER program. Both programs were trained (that is, optimized) using sets of non-redundant structures selected by their respective developers. The REP potential, which was derived in a similar way to Prosa II, used the same non-redundant structure space covered by the EvPs. Thus, the results from the REP potential reflect the impact of using a different structure space with respect to the results from Prosa II. The accuracies of REP and Prosa II are statistically indistinguishable at a 99% confidence cut-off (Table S5 in Additional data file 1). It is important to note that Prosa II, DFIRE Cβ, and EvP were originally developed with the aim of general applicability to any set of protein structure models. Thus, we believe that comparing them using a test set that mimics a large-scale comparative modeling setting is a fair comparison.

The clustering of the structural space and the depth of the MSAs affect the accuracy and coverage of the EvPs. Stricter cut-offs for structural clustering and more permissive cut-offs for MSAs result in more accurate EvPs. This indicates that EvPs are very specific to the structure they represent. However, since a minimum number and diversity of sequences are needed to derive an accurate potential, only those EvPs derived for structures that had more than 50 sequences in the MSA were used in our benchmark (that is, approximately 88% of the representative structures). Increasing the MSA filtering cut-off dramatically lowered the coverage of our method (that is, increasing the sequence identity cut-off from 20% to 40% decreased the coverage by approximately 30%).

The results indicate that the specificity to a given protein structure rather than other properties of the EvPs is what makes them more accurate with respect to other classically derived knowledge-based potentials. In other words, a more specific potential results in more accurate model assessment. However, as for any knowledge-based potential, the derivation of frequencies requires enough sampling for statistical significance. In classically derived potentials, increasing the number of non-redundant folds used during derivation solves the problem. For EvPs, having a sufficient number of diverse sequences in the MSA solves the problem. Thus, the accuracy of the EvPs depends on a fine balance between structure diversity and statistical reliability.

The optimal structure and sequence clustering parameters for deriving reliable EvPs were 90% structural similarity, 90% sequence similarity and a 20% cut-off for the identity between the template sequence and the sequences in the MSA. Although a 20% sequence identity may seem very low for homology detection, all selected sequences for deriving an EvP resulted in a significant PSI-BLAST alignment to the query structure with an e-value smaller than 5 × 10^-4^. Thus, the resulting alignments usually covered a substantial part of the query structure and had short insertions/deletions. Optimal EvPs thus represent a very restricted subset of the structural space covered by a diverse set of sequences, which most probably adopt the fold corresponding to the selected structural space. This result agrees with the notion that protein folding, at least for one-domain proteins, is largely determined by the topology of a protein's native structure rather than its sequence-specific details [23,24]. We propose that the use of homologous sequences for deriving an EvP capture the conservation of sequence-unspecific features, such as the solvent-accessible area or the folding φ-values (that is, the effect of a mutation stabilizing the folding transition state compared to stabilizing the native conformation). Thus, EvPs may be able to effectively encode for both structure and sequence based features when assessing the accuracy of protein structure models.

The results from the CON potential, which accounts for the linear sum of all calculated frequencies used to derive individual EvPs, show that the accuracy achieved by the EvPs is not a simple and direct consequence of maximizing the extraction of information from all available sequences and structures, but rather a result of exploiting the information from only those sequences that belong to a restricted structural space. Indeed, the use of less specific structure space resulted in a 9.5% decrease in the TPR with respect to the EvPs (Table 4). However, using the homologous sequence space to all structures in the non-redundant set of structures resulted in an accuracy increase with respect to other potentials such as REP, Prosa II and DFIRE (Table 4). This suggests that not only the thermodynamic features, important for protein structure stability, are captured by this methodology but also key sequence determinants necessary for fast protein folding.

EvPs are specially suited for the assessment of protein structure models based on comparative approaches where the template used for modeling is known. Template-free methods produce protein structure models based on small fragments of known structure or without the use of templates. Therefore, to apply our method in such cases, it is necessary to use a sequence-based search method for selecting the most appropriate EvP to assess the accuracy of a given model. The results indicate that the use of BLAST or PSI-BLAST to select an EvP decreases their AUC by 2.0% and 1.8%, respectively. However, selecting a representative EvP by BLAST or PSI-BLAST still results in more accurate assessment of models than the REP potential (that is, 1.6% and 1.8% larger AUC, respectively). Most importantly, compared to the REP potential, using the EvP potentials selected by BLAST or PSI-BLAST reduced the FPR by 4.4 and 3.4%, respectively. This demonstrates that EvPs are not limited to models built from comparative approaches and can also be implemented in a general pipeline for protein structure assessment and prediction.

## Conclusion

The results from our work indicate that the use of homologous sequences allows the derivation of specific knowledge-based potentials for protein structure model assessment. In contrast to standard knowledge-based potentials, which are usually derived from a non-redundant set of native structures, the EvPs could also be capturing sequence features that may affect the folding kinetics of a protein. EvPs outperformed other tested potentials with approximately 7-11% higher accuracy, approximately 12-20% higher TPR, and approximately 4% lower FPR. Such an increase in sensitivity and specificity can have a significant impact on large-scale automated protein structure prediction of genomes, which could result in the correct assessment of the folds of more than 500,000 extra models (that is, increase the TPR) and the discarding of about 160,000 extra incorrect models (that is, decrease the FPR). The novelty of EvPs and the conceptually different approach for deriving them could have a broad impact on protein structure prediction and design. Both fields have now reached a mature state and comprise several methods that can produce fairly accurate models for about half of the sequences of an average genome [25,26]. Thus, the development of new methods that increase the accuracy and usefulness of models from widely used computational approaches could prove very useful to thousands of researchers worldwide.

## Materials and methods

### Non-redundant set of protein structures

The redundancy in the PDB database (June 2005) was filtered to a representative list such that the MAMMOTH alignment [27] of any two chains in the list fails at least one of the following four cut-offs: a minimum of 90% sequence identity; a minimum of 90% of Cα atoms aligned within 4 Å; a maximum of 1 Å Cα root mean square deviation; and a maximum of a 50 residue difference in length. Each non-redundant chain represents all other PDB chains in the initial list that pass the cut-offs listed above for all pairwise comparisons within the group; where possible, the representative was picked by maximizing its resolution. Additionally, obsolete PDB entries as well as entries with missing atoms were removed from the initial set, resulting in a final list of 22,732 protein chains. To assess the impact of the PDB redundancy on the accuracy of the EvPs in model assessment, the final representative set of chains was further clustered by varying the sequence identity and structure similarity cut-offs (Table S1 in Additional data file 1).

### Multiple sequence alignments

A MSA for each of the 22,732 non-redundant PDB chains was built using PSI-BLAST (version 2.2.10) [28] to search against the NCBI *nr *database (June 2005). The search was performed without filtering out compositionally biased segments, running for up to 5 iterations, and including up to 100,000 sequence hits with an e-value smaller than 5 × 10^-4^. All other PSI-BLAST parameters were set to their default values. Removing those protein chains that aligned with less than 20%, 40% or 60% sequence identity to the query protein further filtered the MSAs. Finally, all filtered MSAs with 50 or more sequences were used for deriving EvPs (Table S1 in Additional data file 1).

### Sequence weighting

A position-based sequence weighting that assigns low weights to over-represented sequences and high weights to unique sequences was used to compensate for non-uniform distribution of the homologous protein sequences in a MSA [29]. The sequence weights *W*_*j *_were calculated as:

$$W_{j}=\sum_{i}\frac{1}{r_{i}\cdot n_{i,j}}$$

where *r*_*i *_is the number of different residue types at position *i*, and *n*_*i*,*j *_is the frequency of occurrence of the residue type in position *i *and sequence *j *with respect to all residues in position *i*.

### Derivation of knowledge-based potentials

**Figure 4:**
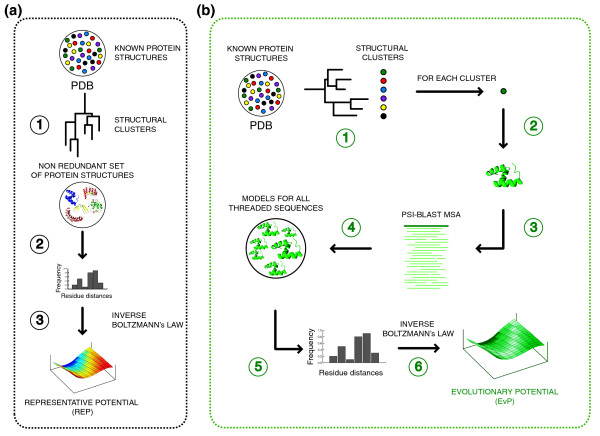
EvP and REP derivation protocols. **(a) **The REP potential was built in a three-step process to: step 1, generate a non-redundant set of protein structures from the PDB database; step 2, calculate all residue-residue distance frequencies within each of the representative chains from step 1; and step 3, derive a knowledge-based potential using the inverse Boltzmann law to transform the raw frequencies into pseudo-energy terms. **(b) **The EvPs were built in a six-step process to: step 1, generate a non-redundant set of protein structures from the PDB database; step 2, select each of the representative chains as query structures; step 3, calculate a MSA using the PSI-BLAST program; step 4, thread all homologous sequences into the query structure using the sequence-based alignment from the previous step; step 5, calculate all residue-residue distance frequencies; and step 6, derive a knowledge-based potential using the inverse Boltzmann law to transform the raw frequencies into pseudo-energy terms.

Two different types of knowledge-based potentials were derived in this work: a representative distance-dependent potential (REP), used as a baseline to benchmark the impact of our new approach, and a series of structure specific distance-dependent potentials here termed EvPs. The unique difference between the REP and the EvP potentials was the input structural space selected for their derivation as well as the use of sequence information. On the one hand, the REP potential was calculated from a set of 22,732 non-redundant protein structures (Figure 4a) following the approach commonly used to derive distance-dependent potentials [7,19,30-35]. On the other hand, for 20,008 of the 22,732 non-redundant protein structures (that is, structures with more than 50 homologous sequences in their MSA), an EvP was calculated using the sequence variability in a set of homologous sequences to the selected structure (Figure 4b). Each EvP was derived by virtually threading all homologous sequences in the MSA into the selected structure, which was used as a guide for the replacement of the amino-acid type at each position. Thus, one can say that the 20,008 EvPs encode the sequence variation observed in the MSA for each of the non-redundant structures. Briefly, the threading approach implemented for deriving EvPs followed three steps: first, collect all pairwise alignments between the selected structure and its homologous sequences in the MSA; second, using each pairwise alignment as a guide, replace the amino-acid type in the selected structure by the one in the homologous sequence; and third, for a gapped position keep the original residue in the selected structure. Two variations of this protocol were also tested, which included the removal of residues in the structure aligned to a gap and the renumbering of the template residues (that is, affecting the sequence separation value of the statistical potential). The tested protocols showed no statistical differences between the resulting EvPs (Table S6 in Additional data file 1). The counting of residue-residue interactions for deriving an EvP was proportional to the sequence weight that accounts for redundancy within the MSA.

In contrast to the REP, where the non-redundant set of protein structures constituted its training set, there was not a single and unique training set for deriving an EvP. The training sets used in EvPs were the actual multiple sequence alignments specific for each selected structure.

In addition to the REP and the EvPs, a single consensus potential (CON) was derived using the sum of observed interaction frequencies from each of the 20,008 individual EvPs. Thus, the CON potential encodes the structural space encompassed by the non-redundant set of structures as well as the sequence space occupied by their homologous sequences.

All potentials derived in this work were calculated using our previously optimized parameters for model assessment [7]. Briefly, the potentials used Cα and Cβ atoms as interaction centers, distinguished between all 20 standard residue types, had a maximal distance range of 15 Å distributed in 30 bins of 0.5 Å each, and accounted for the sequence separation of the interacting atom pairs. Local interactions were considered independently using sequence separations of 2, 3, 4, 5, 6, 7 and 8 residues and non-local interactions were considered by grouping into a single term the interactions with sequence separations larger than or equal to 9 residues.

### Z-scores

Energy Z-scores were calculated based on the protein model energy, the mean and the standard deviation of the knowledge-based potential energy of 1,000 random sequences with the same amino acid composition and structure of the protein model, as previously described [7].

### Model assessment protocol

An EvP was calculated for each of the non-redundant chains in the PDB and represented a given set of similar structures. Thus, the selection of an EvP for assessing the accuracy of a given model could have an impact on the final accuracy of our method. Several protocols were implemented and tested to assess such an impact.

#### Template-based selection

The template structure used to build the model was obtained from the corresponding sequence-structure alignment used during the modeling. Then, the EvP representing the template's structural cluster was used to evaluate the accuracy of the model.

#### Template-free selection

In order to assess the impact of the EvP selection for template-free models, the PSI-BLAST and BLAST algorithms were used with default values to detect the closest match between the sequence of the model and our database of EvPs.

#### Random selection

The so-called random potential (RND) was calculated by randomly selecting one of the 20,008 EvPs to assess the accuracy of a given model.

To avoid biased results, the EvP derived for the target structure was removed prior to EVP selection in all three protocols. However, it is important to note that it is not certain, even conceptually, that rigorous testing of a method should not rely on structures similar or identical to those from which the potentials were derived. In practice, statistical potentials are to be used in model assessment of comparative models that, by construction, are similar to known protein structures. Therefore, all of the known protein structures are legitimate sources for deriving any of the statistical potentials used in practical model assessment, including those known structures that happen to be related to the assessed model.

### Test set of comparative models

The evaluation of the EvPs for model assessment was based on an initial set of 9,645 structural models divided into 3,375 correct and 6,270 incorrect models [7,22]. A correct model was defined as a model for which at least 30% of the Cα atoms superimposed within 3.5 Å with those of the real structure, and thus is based on proper fold assignment and a relatively accurate sequence/structure alignment. Incorrect models (that is, superimposing less than 15% of the Cα atoms within 3.5 Å) were built using a wrong fold or based on the correct fold, but containing a large fraction of misalignments. Thus, the test set of protein structure models, which was the result of a large-scale comparative modeling of the complete PDB [22], represented the known protein structural space. This set of comparative models has been previously and extensively used to benchmark methods of model assessment [7,17,22,36,37].

To be able to fairly compare all potentials, the initial test set was reduced to 1,877 correct and 2,567 incorrect models, which corresponded to those for which an EvP could be derived for all clustering cut-offs (Table S1 in Additional data file 1). Since an EvP cannot be reliably derived for representative structures with less than 50 homologous sequences [7], a large fraction of models did not have a derived EvP for their corresponding template structures in the CLS-90-90_MSA-60 cluster. However, an EvP at CLS-90-90 and MSA-20, which corresponds to the most accurate knowledge-based potential (Results), could be calculated for 96.4% (3,253) and 94.8% (5,942) of correct and incorrect models in the test set, respectively.

All potential scores, the models for the two datasets used in this work as well as the EvPs are available for download at [38].

### Benchmarking criteria

The accuracy of the knowledge-based potentials was evaluated by means of the maximal accuracy (ACC) and the AUC, which were calculated from a receiver operating characteristic (ROC) curve [39] using correct models as positive instances and incorrect models as negative instances. A ROC curve is obtained by plotting the FPR (that is, fraction of incorrect models assessed as correct) against the corresponding TPR (that is, fraction of correct models assessed as correct) for all possible cut-offs on the energy Z-score. The AUC, a threshold independent measure, is considered a robust indicator of a classifier quality given its independence from the selected threshold and its correlation with the probability of the classifier error [39]. The optimal classification threshold leading to the maximal ACC is also reported for each tested potential.

### Other benchmarked methods

Two widely used knowledge-based potentials for error detection in protein structure models were also evaluated to provide an additional and objective reference frame for evaluating the accuracy of the EvPs. First, the Prosa II program [4,20,21], derived from a set of non-redundant structures, calculates an energy score and a Z-score for an input model. Second, the DFIRE program [19], derived by using a distance-scaled finite ideal-gas as reference state, calculates an energy score for a model. The final DFIRE Z-scores were calculated using the procedure described above. Both programs, Prosa II and DFIRE, were locally run using their respective default parameters.

### Statistical significance of the differences between the evaluated potentials

The statistical significance of the observed differences between two potentials used as binary classifiers was evaluated by a non-parametric test that accounts for the correlation of the ROC curves [40]. This test takes advantage of the equality between the Mann-Whitney U-statistic and the AUC when computed by the trapezoidal rule for comparing two distributions. A chi-square statistic computes the significance (*p*-value) of the difference between the AUC measured for the two classifiers. The results corresponding to the statistical comparisons are reported in the Additional data file 1 (Tables S1, and S3-S5).

## Abbreviations

ACC, accuracy; AUC, area under the curve; CON, consensus EvP potential; EvP, evolutionary potential; FPR, false positive rate; MSA, multiple sequence alignment; OC, optimal cut-off; REP, representative potential; ROC, receiver operating characteristic; TPR, true positive rate;.

## Authors' contributions

MAM-R and FM conceived the idea and supervised the project; AP, FM and MAM-R planned and performed the research as well as analyzed its results; all authors wrote and approved the final manuscript.

## Additional data files

The following additional data are available with the online version of this paper. Additional file 1 contains Tables S1-S6.

## Supplementary Material

Additional data file 1 Tables S1, S3, S4 and S5 show the results from a statistical analysis of the accuracy differences between the tested statistical potentials. Table S2 shows information about the tested EvPs. Table S6 shows the results of EvPs upon changes in the threading parameters. Click here for file

## References

[B1] Marti-Renom MA Stuart AC Fiser A Sanchez R Melo F Sali A Comparative protein structure modeling of genes and genomes. Annu Rev Biophys Biomol Struct 2000 29 291 325 10.1146/annurev.biophys.29.1.291 10940251

[B2] Marti-Renom MA Madhusudhan MS Sali A Alignment of protein sequences by their profiles. Protein Sci 2004 13 1071 1087 10.1110/ps.03379804 15044736 PMC2280052

[B3] Mackerell AD Jr Empirical force fields for biological macromolecules: overview and issues. J Comput Chem 2004 25 1584 1604 10.1002/jcc.20082 15264253

[B4] Sippl MJ Boltzmann's principle, knowledge-based mean fields and protein folding. An approach to the computational determination of protein structures. J Comput Aided Mol Des 1993 7 473 501 10.1007/BF02337562 8229096

[B5] Miyazawa S Jernigan RL Residue-residue potentials with a favorable contact pair term and an unfavorable high packing density term, for simulation and threading. J Mol Biol 1996 256 623 644 10.1006/jmbi.1996.0114 8604144

[B6] Domingues FS Koppensteiner WA Jaritz M Prlic A Weichenberger C Wiederstein M Floeckner H Lackner P Sippl MJ Sustained performance of knowledge-based potentials in fold recognition. Proteins 1999 Suppl 3 112 120 10.1002/(SICI)1097-0134(1999)37:3+<112::AID-PROT15>3.0.CO;2-R 10526359

[B7] Melo F Sanchez R Sali A Statistical potentials for fold assessment. Protein Sci 2002 11 430 448 10.1110/ps.25502 11790853 PMC2373452

[B8] McGuffin LJ Jones DT Improvement of the GenTHREADER method for genomic fold recognition. Bioinformatics 2003 19 874 881 10.1093/bioinformatics/btg097 12724298

[B9] Melo F Feytmans E Assessing protein structures with a non-local atomic interaction energy. J Mol Biol 1998 277 1141 1152 10.1006/jmbi.1998.1665 9571028

[B10] Zhou H Zhou Y Distance-scaled, finite ideal-gas reference state improves structure-derived potentials of mean force for structure selection and stability prediction. Protein Sci 2002 11 2714 2726 10.1110/ps.0217002 12381853 PMC2373736

[B11] Tsai J Bonneau R Morozov AV Kuhlman B Rohl CA Baker D An improved protein decoy set for testing energy functions for protein structure prediction. Proteins 2003 53 76 87 10.1002/prot.10454 12945051

[B12] Zhu J Zhu Q Shi Y Liu H How well can we predict native contacts in proteins based on decoy structures and their energies? Proteins 2003 52 598 608 10.1002/prot.10444 12910459

[B13] Lu H Skolnick J A distance-dependent atomic knowledge-based potential for improved protein structure selection. Proteins 2001 44 223 232 10.1002/prot.1087 11455595

[B14] Wallner B Elofsson A Can correct protein models be identified? Protein Sci 2003 12 1073 1086 10.1110/ps.0236803 12717029 PMC2323877

[B15] Park B Levitt M Energy functions that discriminate X-ray and near native folds from well-constructed decoys. J Mol Biol 1996 258 367 392 10.1006/jmbi.1996.0256 8627632

[B16] Park BH Huang ES Levitt M Factors affecting the ability of energy functions to discriminate correct from incorrect folds. J Mol Biol 1997 266 831 846 10.1006/jmbi.1996.0809 9102472

[B17] Melo F Sali A Fold assessment for comparative protein structure modeling. Protein Sci 2007 16 2412 2426 10.1110/ps.072895107 17905832 PMC2211691

[B18] Melo F Sanchez R Sali A Statistical potentials for fold assessment. Protein Sci 2002 11 430 448 10.1110/ps.25502 11790853 PMC2373452

[B19] Zhou H Zhou Y Distance-scaled, finite ideal-gas reference state improves structure-derived potentials of mean force for structure selection and stability prediction. Protein Sci 2002 11 2714 2726 10.1110/ps.0217002 12381853 PMC2373736

[B20] Sippl MJ Weitckus S Detection of native-like models for amino acid sequences of unknown three-dimensional structure in a data base of known protein conformations. Proteins 1992 13 258 271 10.1002/prot.340130308 1603814

[B21] Sippl MJ Knowledge-based potentials for proteins. Curr Opin Struct Biol 1995 5 229 235 10.1016/0959-440X(95)80081-6 7648326

[B22] Sanchez R Sali A Large-scale protein structure modeling of the *Saccharomyces cerevisiae genome*. Proc Natl Acad Sci USA 1998 95 13597 13602 10.1073/pnas.95.23.13597 9811845 PMC24864

[B23] Plaxco KW Larson S Ruczinski I Riddle DS Thayer EC Buchwitz B Davidson AR Baker D Evolutionary conservation in protein folding kinetics. J Mol Biol 2000 298 303 312 10.1006/jmbi.1999.3663 10764599

[B24] Zarrine-Afsar A Larson SM Davidson AR The family feud: do proteins with similar structures fold via the same pathway? Curr Opin Struct Biol 2005 15 42 49 10.1016/j.sbi.2005.01.011 15718132

[B25] Pieper U Eswar N Davis FP Braberg H Madhusudhan MS Rossi A Marti-Renom M Karchin R Webb BM Eramian D Shen MY Kelly L Melo F Sali A MODBASE: a database of annotated comparative protein structure models and associated resources. Nucleic Acids Res 2006 34 D291 D295 10.1093/nar/gkj059 16381869 PMC1347422

[B26] Kopp J Schwede T The SWISS-MODEL Repository: new features and functionalities. Nucleic Acids Res 2006 34 D315 318 10.1093/nar/gkj056 16381875 PMC1347419

[B27] Ortiz AR Strauss CE Olmea O MAMMOTH (matching molecular models obtained from theory): an automated method for model comparison. Protein Sci 2002 11 2606 2621 10.1110/ps.0215902 12381844 PMC2373724

[B28] Altschul SF Madden TL Schaffer AA Zhang J Zhang Z Miller W Lipman DJ Gapped BLAST and PSI-BLAST: a new generation of protein database search programs. Nucleic Acids Res 1997 25 3389 3402 10.1093/nar/25.17.3389 9254694 PMC146917

[B29] Henikoff S Henikoff JG Position-based sequence weights. J Mol Biol 1994 243 574 578 10.1016/0022-2836(94)90032-9 7966282

[B30] Sippl MJ Calculation of conformational ensembles from potentials of mean force. An approach to the knowledge-based prediction of local structures in globular proteins. J Mol Biol 1990 213 859 883 10.1016/S0022-2836(05)80269-4 2359125

[B31] Jones DT GenTHREADER: an efficient and reliable protein fold recognition method for genomic sequences. J Mol Biol 1999 287 797 815 10.1006/jmbi.1999.2583 10191147

[B32] Samudrala R Moult J An all-atom distance-dependent conditional probability discriminatory function for protein structure prediction. J Mol Biol 1998 275 895 916 10.1006/jmbi.1997.1479 9480776

[B33] Kihara D Lu H Kolinski A Skolnick J TOUCHSTONE: an *ab initio *protein structure prediction method that uses threading-based tertiary restraints. Proc Natl Acad Sci USA 2001 98 10125 10130 10.1073/pnas.181328398 11504922 PMC56926

[B34] Melo F Feytmans E Novel knowledge-based mean force potential at atomic level. J Mol Biol 1997 267 207 222 10.1006/jmbi.1996.0868 9096219

[B35] Shen MY Sali A Statistical potential for assessment and prediction of protein structures. Protein Sci 2006 15 2507 2524 10.1110/ps.062416606 17075131 PMC2242414

[B36] Ferrada E Melo F Nonbonded terms extrapolated from nonlocal knowledge-based energy functions improve error detection in near-native protein structure models. Protein Sci 2007 16 1410 1421 10.1110/ps.062735907 17586774 PMC2206707

[B37] Melo F Marti-Renom MA Accuracy of sequence alignment and fold assessment using reduced amino acid alphabets. Proteins 2006 63 986 995 10.1002/prot.20881 16506243

[B38] Bioinformatics datasets from the Structural Genomics Unit at CIPF http://sgu.bioinfo.cipf.es/datasets/

[B39] Fawcett T ROC Graphs: Notes and Practical Considerations for Data Mining Researchers. HP Labs Tech Report HPL-2003-4 2003 http://www.hpl.hp.com/techreports/2003/HPL-2003-4.pdf

[B40] DeLong ER DeLong DM Clarke-Pearson DL Comparing the areas under two or more correlated receiver operating characteristic curves: a nonparametric approach. Biometrics 1988 44 837 845 10.2307/2531595 3203132

