# Supplementary material for: Evolutionary potentials: structure specific knowledge-based potentials exploiting the evolutionary record of sequence homologs
Source: Genome Biol. 2008 Apr 8;9(4):R68. doi: 10.1186/gb-2008-9-4-r68 (PMC2643939; doi:10.1186/gb-2008-9-4-r68)
Supplement: Additional data file 1 — Tables S1, S3, S4 and S5 show the results from a statistical analysis of the accuracy differences between the tested statistical potentials. Table S2 shows information about the tested EvPs. Table S6 shows the results of EvPs upon changes in the threading parameters. [file gb-2008-9-4-r68-S1.doc]

Additional data file 1

Evolutionary potentials: structure specific knowledge-based potentials exploiting the evolutionary record of sequence homologs

Alejandro Panjkovich^1^, Francisco Melo^1*^ and Marc A. Marti-Renom^2*^

^1^ Departamento de Genética Molecular y Microbiología, Facultad de Ciencias Biológicas, Pontificia Universidad Católica de Chile, Alameda 340, Santiago, Chile.

^2^ Structural Genomics Unit, Bioinformatics Department, Centro de Investigación Príncipe Felipe (CIPF), Av. Autopista del Saler, 16, 46013 Valencia, Spain

* Corresponding Authors:

Francisco Melo

Departamento de Genética Molecular y Microbiología, Facultad de Ciencias Biológicas, Pontificia Universidad Católica de Chile, Alameda 340, Santiago, Chile Tel: +56 2 686 2279; Fax: +56 2 222 55 15; e-mail: [fmelo@bio.puc.cl](mailto:fmelo@bio.puc.cl)

[http://protein.bio.puc.cl](http://protein.bio.puc.cl/)

Marc A. Marti-Renom

Structural Genomics Unit,

Bioinformatics Department, Centro de Investigación Príncipe Felipe (CIPF)

Av. Autopista del Saler, 16, 46013 Valencia, Spain

Tel: +34 96 3289680; fax: +34 96 3289701; email: [mmarti@cipf.es](mailto:mmarti@cipf.es)

[http://sgu.bioinfo.cipf.es](http://sgu.bioinfo.cipf.es/)

*Version:* August 29, 2026

**Table S1. Statistical significance of results from Figure 2A.**

| **CLASSIFIER** | CLS_90-90 | CLS_90-80 | CLS_90-10 | CLS_90-50 | CLS_80-80 | CLS_80-50 | CLS_80-10 |
| --- | --- | --- | --- | --- | --- | --- | --- |
| CLS_90-90 |  | 0.168 | 0.437 | 0.446 | 0.614 | 1.751 | 1.814 |
| CLS_90-80 | 0.001 |  | 0.268 | 0.277 | 0.446 | 1.582 | 1.645 |
| CLS_90-10 | 6.57E-05 | 0.009 |  | 8.51E-05 | 0.177 | 1.314 | 1.377 |
| CLS_90-50 | 5.33E-05 | 0.008 | **0.107** |  | 0.169 | 1.305 | 1.368 |
| CLS_80-80 | 1.2E-06 | 0.001 | **0.260** | **0.285** |  | 1.137 | 1.199 |
| CLS_80-50 | 6.28E-17 | 6.15E-15 | 5.64E-13 | 8.3E-13 | 2.04E-11 |  | 0.063 |
| CLS_80-10 | 3.38E-18 | 6.95E-16 | 1.45E-14 | 2.02E-14 | 5.59E-11 | **0.588** |  |

The sets of EvPs are named upon the clustering parameters (CLS_XX-YY). XX is the minimal structural similarity and YY is the minimal sequence identity used for structural clustering. A minimal sequence identity of 20% between a sequence in the multiple sequence alignment and the representative structure of the cluster was used.

The upper right triangle of the matrix corresponds to the differences between the compared methods in the AUC measure. The lower left triangle of the matrix corresponds to the p-value significance of the difference (cases where the AUC differences are not statistically significant at a confidence level of 95% are shown in bold face).

**Table S2. EvPs tested for fold assessment**

|  | **EvPs** | | **Multiple sequence alignment** | |
| --- | --- | --- | --- | --- |
| **EvP set** | **Potential^1^** | **Calculated ^2^** | **Average number of sequences^3^** | **Average sequence identity^4^ (%)** |
| CLS-90-90_MSA-60 | 22,732 | 7,098 | 1,217 | 77.0 |
| CLS-90-90_MSA-40 | 22,732 | 13,520 | 1,639 | 59.2 |
| CLS-90-90_MSA-20 | 22,732 | 20,054 | 2,591 | 36.9 |
| CLS-90-80_MSA-20 | 20,713 | 18,294 | 2,539 | 36.6 |
| CLS-90-50_MSA-20 | 20,288 | 17,899 | 2,515 | 36.5 |
| CLS-90-20_MSA-20 | 20,204 | 17,831 | 2,517 | 36.5 |
| CLS-90-10_MSA-20 | 20,182 | 17,817 | 2,518 | 36.5 |
| CLS-80-80_MSA-20 | 14.460 | 12,650 | 2,280 | 36.0 |
| CLS-80-50_MSA-20 | 13,206 | 11,475 | 2,059 | 35.8 |
| CLS-80-10_MSA-20 | 12,379 | 10,730 | 2,065 | 35.8 |

The sets of EvPs are named upon the clustering parameters (CLS_XX-YY) and the multiple sequence alignment filters (MSA_ZZ) used to derive them (see Methods). XX is the minimal structural similarity and YY is the minimal sequence identity used for structural clustering. ZZ is the minimal sequence identity shared between a sequence in the multiple sequence alignment and the representative structure of the cluster.

^1^Number of potential EvPs given the set of clustering cut-offs XX and YY.

^2^Number of calculated EvPs with more than 50 homologous sequences in the multiple sequence alignment.

^3^Average number of sequences in the multiple sequence alignments.

^4^Average sequence identity percentage between the representative chain and its homologous sequences in the multiple sequence alignment.

**Table S3. Statistical significance of results from Figure 2B.**

| **CLASSIFIER** | MSA_20 | MSA_40 | MSA_60 |
| --- | --- | --- | --- |
| MSA_20 |  | 0.493 | 1.823 |
| MSA_40 | 1.3E-08 |  | 1.330 |
| MSA_60 | 1.15E-22 | 1.68E-20 |  |

The sets of EvPs are named upon the multiple sequence alignment filters (MSA_ZZ) used to derive them. ZZ is the minimal sequence identity shared between a sequence in the multiple sequence alignment and the representative structure of the cluster. A minimal structural similarity of 90% and a minimal sequence identity of 90% were used for structural clustering.

The upper right triangle of the matrix corresponds to the differences between the compared methods in the AUC measure. The lower left triangle of the matrix corresponds to the p-value significance of the difference.

**Table S4. Statistical significance of results from Figure 3.**

| **CLASSIFIER** | EvP  (CLUSTER) | EvP  (PSI-BLAST) | EvP  (BLAST) | CON | REP | RND |
| --- | --- | --- | --- | --- | --- | --- |
| EvP (CLUSTER) |  | 1.736 | 1.961 | 2.393 | 3.566 | 27.558 |
| EvP (PSI-BLAST) | 1.12E-14 |  | 0.225 | 0.658 | 1.829 | 25.822 |
| EvP (BLAST) | 2.35E-17 | **0.128** |  | 0.431 | 1.605 | 25.597 |
| CON | 5.32E-24 | 0.034 | **0.170** |  | 1.172 | 25.165 |
| REP | 2.14E-40 | 1.84E-09 | 1.99E-07 | 9.28E-07 |  | 23.993 |
| RND | 1.61E-273 | 6.39E-235 | 4.17E-233 | 1.14E-230 | 4.24E-221 |  |

The upper right triangle of the matrix corresponds to the differences between the compared methods in the AUC measure. The lower left triangle of the matrix corresponds to the p-value significance of the difference (cases where the AUC differences are not statistically significant at a confidence level of 95% are shown in bold face).

**Table S5. Statistical significance of results from Figure 4.**

| **CLASSIFIER** | EvP (CLUSTER) | REP | Prosa II | DFIRE Cβ |
| --- | --- | --- | --- | --- |
| EvP (CLUSTER) |  | 3.566 | 4.140 | 6.730 |
| REP | 2.14E-40 |  | 0.575 | 3.164 |
| Prosa II | 1.43E-46 | 0.015 |  | 2.589 |
| DFIRE Cβ | 2.67E-61 | 5.16E-16 | 1.07E-10 |  |

The upper right triangle of the matrix corresponds to the differences between the compared methods in the AUC accuracy measure. The lower left triangle of the matrix corresponds to the p-value significance of the difference.

**Table S6. Results from the three different threading protocols.**

| **Name** | **Procedure** | **AUC (%)** | **ACC (%)** |
| --- | --- | --- | --- |
| EvP type 1 | Standard protocol (steps i to iii) | 99.2 | 97.4 |
| EvP type 2 | Remove template residues in gap positions | 99.3 | 97.5 |
| EvP type 3 | Remove residues and renumber (k) | 99.2 | 97.5 |

**Supplementary data**

Comparative protein structure models and all calculated data described in the manuscript are available for download at: <http://sgu.bioinfo.cipf.es/datasets>.
